# Ultrasound evaluation of inguinoscrotal pain: an imaging-based review for the ultrasonographer

**DOI:** 10.1590/0100-3984.2016.0175

**Published:** 2018

**Authors:** Eduardo Kaiser Ururahy Nunes Fonseca, Milena Rocha Peixoto, Francisco de Assis Cavalcante Júnior, Antonio Rahal Júnior, Miguel José Francisco Neto, Marcelo Buarque de Gusmão Funari

**Affiliations:** 1 MD, Resident in the Imaging Department of the Hospital Israelita Albert Einstein, São Paulo, SP, Brazil.; 2 MD, Radiologist in the Imaging Department of the Hospital Israelita Albert Einstein, São Paulo, SP, Brazil.; 3 MD, PhD, Radiologist and Coordinator of the Ultrasound Group in the Imaging Department of the Hospital Israelita Albert Einstein, São Paulo, SP, Brazil.; 4 MD, PhD, Radiologist and Coordinator of the Imaging Department of the Hospital Israelita Albert Einstein, São Paulo, SP, Brazil.

**Keywords:** Hernia, inguinal, Inguinal canal, Orchitis, Fournier gangrene, Ultrasonography

## Abstract

Emergencies involving the inguinal region and scrotum are common and can be caused by a plethora of different causes. In most cases, such conditions have nonspecific symptoms and are quite painful. Some inguinoscrotal conditions have high complication rates. Early and accurate diagnosis is therefore imperative. Ultrasound is the method of choice for the initial evaluation of this vast range of conditions, because it is a rapid, ionizing radiation-free, low-cost method. Despite the practicality and accuracy of the method, which make it ideal for use in emergency care, the examiner should be experienced and should be familiarized with the ultrasound findings of the most common inguinoscrotal diseases. On the basis of that knowledge, the examiner should also be able to make an accurate, direct, precise report, helping the emergency room physician make decisions regarding the proper (clinical or surgical) management of each case. Here, we review most of the inguinoscrotal conditions, focusing on the imaging findings and discussing the critical points for the appropriate characterization of each condition.

## INTRODUCTION

Pain in the scrotum and groin can have a wide range of clinical presentations. The pain can range from mild to lancinating, and patients with such pain can present with normal complete blood counts or with leukocyte counts indicative of severe sepsis. Rapid and accurate diagnosis is essential for differentiating between more severe conditions and less severe ones, as well as for determining which patients can be released with medication and which ones must be hospitalized or require emergency surgical intervention. Making a rapid and accurate diagnosis can also allow early intervention for complications such as the progression from testicular torsion to permanent injury and atrophy.

The objective of this study was to discuss the technique for ultrasound examination of the inguinal region and scrotum, as well as to identify the most common causes of inguinoscrotal pain seen in the emergency room. We also list the specific imaging characteristics that should be known to ultrasound examiners.

## TECHNIQUE

**Figure 1:**
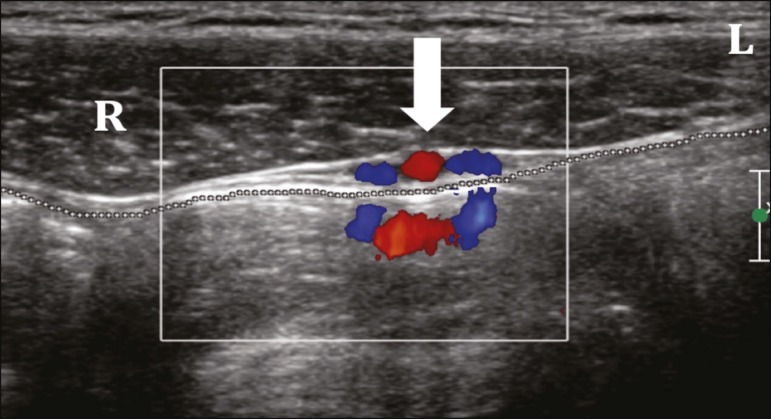
Normal anatomy of the inguinal region. Axial plane. The rectus abdominis muscle (R), oblique abdominal muscles (L), epigastric vessels (arrow), and peritoneal fat interface (dotted line) with no signs of herniation.

**Figure 2:**
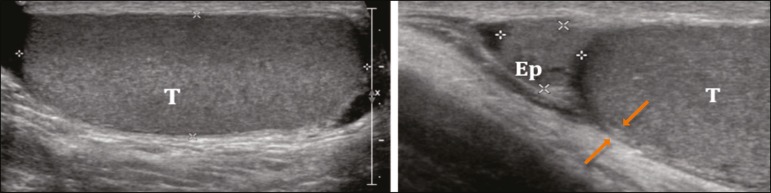
Normal anatomy of the scrotum. The testes (T) show homogeneous granular echogenicity and the testicular mediastinum is seen as a hyperechogenic linear band at the center of each testis. The tunica albuginea appears as a hyperechogenic line around the testicles (demarcated between the arrows), usually with small amount of anechoic fluid between its layers. The head of the epididymis (Ep) can be clearly seen in the sagittal plane, resting on the testicle and with similar echogenicity.

The structures of the inguinal region are superficial and can be evaluated well with linear transducers at 10 MHz or at lower frequencies (7 MHz) in more obese patients. Figure 1 shows the normal anatomy of the inguinal region in the axial plane. The Valsalva maneuver is essential and a critical component of the examination of the region, allowing dynamic evaluation, especially in cases of suspected hernia, because hernias can disappear completely when the patient is at rest, making them difficult to detect^(^^1^^,^^2^^)^. The normal aspect of the scrotum is shown in Figure 2.

## IMAGING FINDINGS

### Hernias

Hernias occur at the weakest points in the abdominal wall, through which the vessels can push and the testes can migrate. They are classified as indirect or direct, depending on their origin in relation to the inferior epigastric vessels.

**Figure 3:**
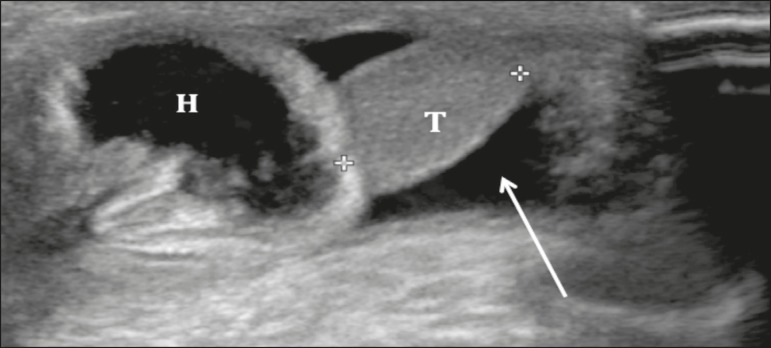
Indirect inguinal hernia. Hernia sac (H) penetrating the scrotum through the inguinal canal. (T, testis).

**Indirect inguinal hernias** - Indirect inguinal hernias are seen protruding anteriorly toward the transducer, originating from an area lateral to the inferior epigastric vessels (Figure 3). They are congenital and are more common in men, in whom their occurrence is due to the persistence of a patent processus vaginalis. In women, the cause is the delayed closure or non-closure of the canal of Nuck^(^^1^^-^^3^^)^.

**Figure 4:**
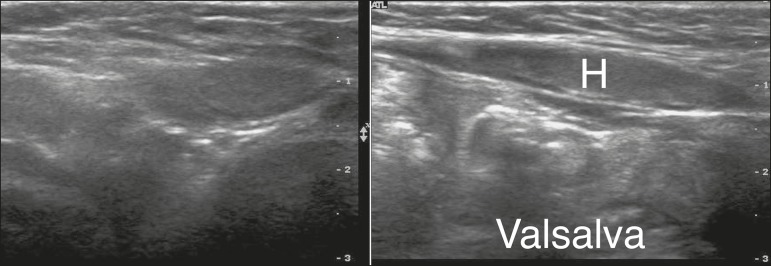
Direct inguinal hernia. Increased hernia content (H) after a Valsalva maneuver.

**Direct inguinal hernias** - Direct inguinal hernias originate medial to the inferior epigastric vessels, in Hasselbach's triangle, which can be evaluated in images of the area superior to the inguinal canal (Figure 4). They are usually acquired, denoting weakness of the transverse fascia. Incarcerated and strangulated hernias are less common because the width of their neck is usually greater than is their depth.

**Femoral hernias** - Femoral hernias also belong to the inguinal group of hernias, due to the proximity of their sites of presentation. More common in females, femoral hernias pass through the femoral canal, therefore being located medial to the femoral vein, and insinuate themselves into the superomedial portion of the thigh^(^^3^^)^.

The vast majority of hernias detected by ultrasound do not contain intestinal loops, comprising only adipose tissue. Incarcerated hernias are those that are not reducible. Possible complications of the incarceration of intestinal loops in hernias include complete or partial obstruction and strangulation. Incarcerated hernias with vascular content are classified as strangulated. On ultrasound, they frequently do not present vascularity on the Doppler flow study, some cases showing parietal thickening and loss of peristalsis if they contain intestinal loops^(^^1^^,^^2^^)^. In such cases, emergency surgery is indicated.

### Spermatic cord torsion

Cases of acute scrotum should, until proven otherwise, be considered attributable to spermatic cord torsion. Such torsion, which can be extravaginal or intravaginal, accounts for a third of all cases of acute scrotum. Extravaginal torsion affects newborns in the first days of life, during the final stage of fixation of the testis. Intravaginal torsion is more common, occurring in older children and adults, its incidence peaking at puberty. Failure of the tunica albuginea during fixation results in the so-called "bell clapper" deformity, which often occurs in both testes, requiring bilateral orchidopexy to avoid contralateral torsion.

**Figure 5:**
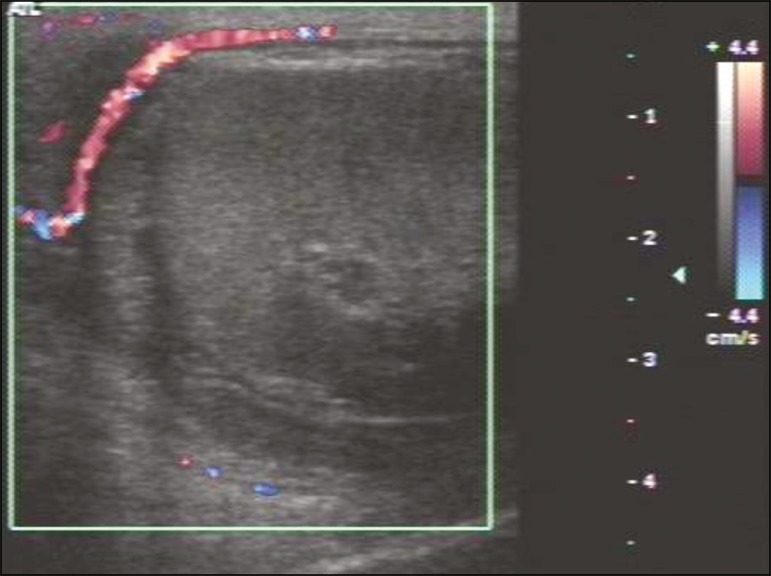
Hypoechoic, heterogeneous, rounded testis. Absence of intratesticular flow on the Doppler flow study.

The testis can be elevated and fixed, with the epididymis in a medial position. Ultrasound in B mode can reveal entanglement of the spermatic cord, similar to a whirl. The persistence of testicular echogenicity indicates that the testis remains viable, and it is imperative that the surgery be performed immediately. Color Doppler ultrasound of the scrotum confirms the diagnosis of torsion. In cases of torsion that is partial (less than 360°), spectral Doppler ultrasound can be of great value in identifying lower diastolic flow in the affected testis than in the contralateral testis, denoting resistance to blood flow in the former. In addition to being minimally invasive and affordable, spectral Doppler ultrasound shows the anatomy of the spermatic cord and the blood flow, which in cases of twisting is reduced or absent^(^^4^^)^, as shown in Figure 5.

### Torsion of the appendix testis

The appendix testis can also be the cause of acute testicular pain, mimicking testicular torsion. The characteristic blue dot sign is seen in only 20% of patients with torsion of the appendix testis, and ultrasound is fundamental in making the differential diagnosis. When the appendix testis is twisted, it will appear enlarged, with variable echogenicity, and asymmetry with the contralateral appendix testis is a good comparative parameter.

**Figure 6:**
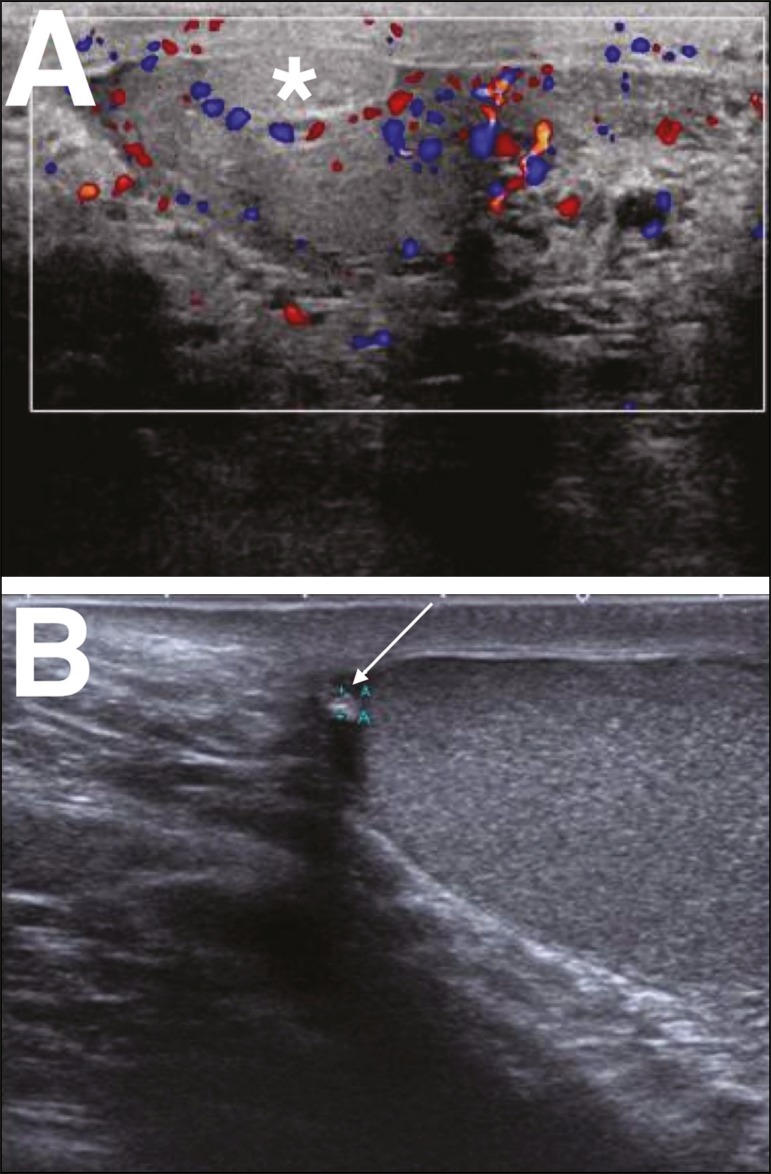
**A:** Torsion of the appendix testis. Note the enlargement of the appendix testis (asterisk), showing no flow on the Doppler flow study. The adjacent testis presents only reactive hyperemia, without alteration of its echotexture. **B:** Chronic torsion of the appendix testis. Note the increased echogenicity and decreased size of the appendix testis (arrow).

A Doppler flow study will show little or no vascularity in the twisted appendix, with possible hyperemia of adjacent tissues. More importantly, there will be no changes in the ipsilateral testis (Figure 6), which rules out the differential diagnosis of spermatic cord torsion^(^^5^^)^. Making the differential diagnosis with spermatic cord torsion is an important part of the management of these cases: the torsion of the appendix testis can be managed with symptomatic treatment, whereas spermatic cord torsion is a urological emergency, requiring immediate surgery to save the affected testis. In chronic cases, the appendix testis can detach and calcify, generating a freely mobile calcified body known as a scrotolith or scrotal pearl.

### Epididymitis

The most common cause of acute scrotal pain in men is epididymitis, the incidence of which peaks between the fifth and sixth decades of life. The pain is initially insidious and increases after 24-48 h. Epididymitis often results from an infection in the lower urinary tract, the most common etiological agent being *Escherichia coli*. In young men, epididymitis is typically a sexually acquired disease occurring from the second to the fourth decades of life, the main causative pathogens being *Chlamydia trachomatis* and *Neisseria gonorrhoeae*.

In acute epididymitis, ultrasound shows thickening and enlargement of the epididymis, initially affecting the caudal portion but potentially affecting the entire organ. The echogenicity is often diminished, and the echotexture is heterogeneous. Reactive hydrocele and cutaneous thickening are common. The effects can extend to the testis, which will produce a hypoechoic area with increased vascularity on a Doppler flow study. In some cases, notably in patients with the mumps, orchitis can occur in the absence of epididymitis.

**Figure 7:**
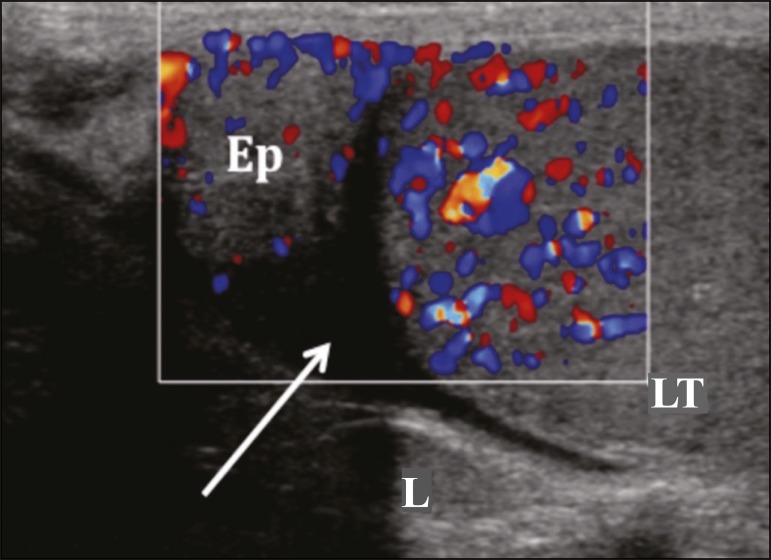
Heterogeneous testis and epididymis, both showing enlargement and hypervascularity on the Doppler flow study. Note the small reactive hydrocele (arrow). (Ep, epididymis; LT, left testis; L, left).

**Figure 8:**
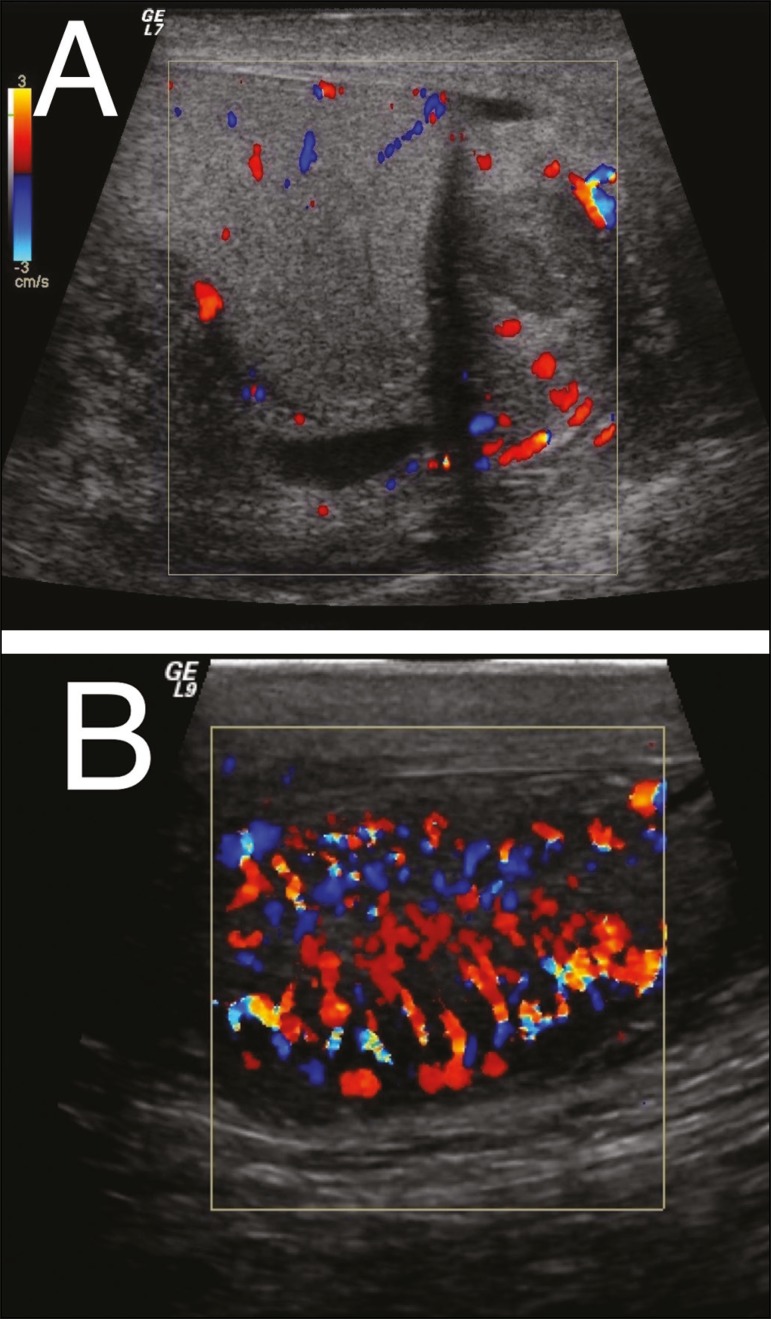
**A:** Heterogeneous, enlarged epididymis, showing hypervascularity on the Doppler flow study. **B:** Heterogeneous, enlarged testis, showing diffuse hypervascularity on the Doppler flow study.

In cases of uncomplicated orchitis, spectral Doppler ultrasound can reveal increased diastolic flow. If orchitis is not treated promptly, the entire testis can be affected, becoming hypoechoic and enlarged. Testicular edema can cause a secondary increase in pressure, with a consequent increase in the risk of venous infarction and hemorrhage. In such cases, a reduction in the diastolic flow seen on spectral Doppler ultrasound serves as a warning sign, denoting resistance to venous blood flow and the possibility of testicular infarction. In addition to infarction, the complications of epididymitis include abscess and pyocele^(^^4^^)^, as depicted in Figures 7 and 8.

### Thrombosis of the pampiniform plexus

**Figure 9:**
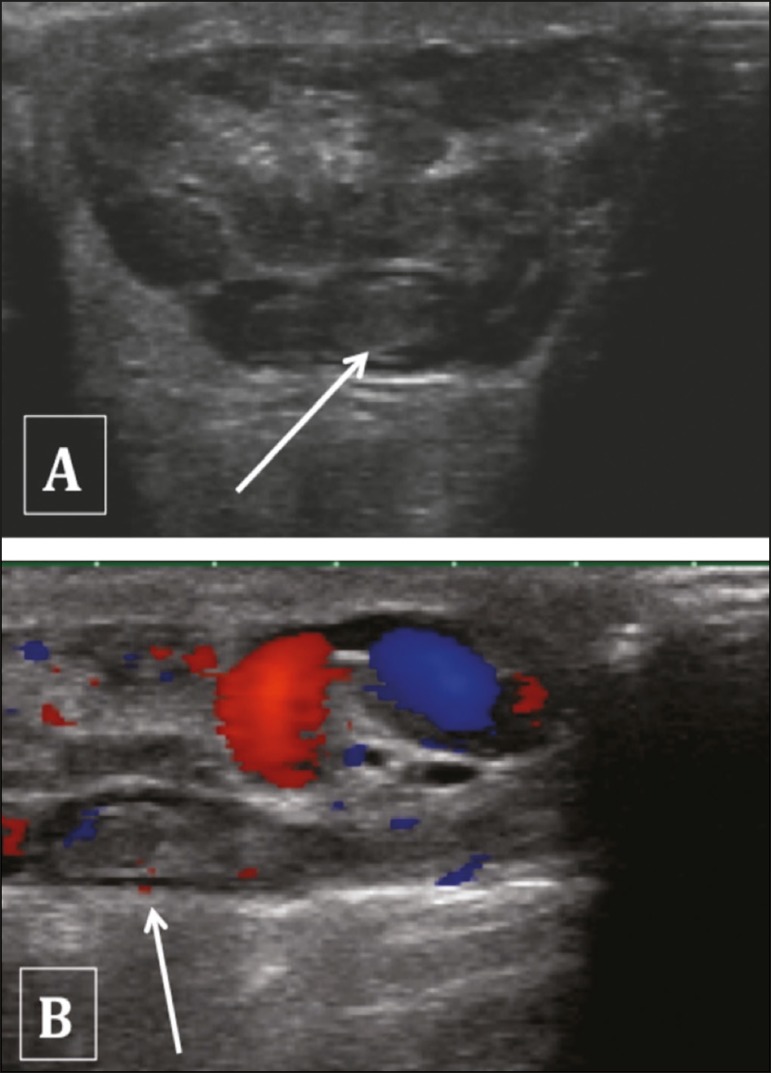
Partial thrombosis of the pampiniform plexus. Note the hypoechoic material in the vascular lumen and the absence of vascularization on the Doppler flow study (arrows).

Spontaneous thrombosis of the pampiniform plexus is rare and difficult to diagnose, with a clinical profile similar to those of other causes of scrotal pain. It is typically associated with intense physical exertion, which leads to an increase in intra-abdominal pressure and a reduction in the venous return. The ultrasound findings are similar to those of varicocele-that is, the vessels of the pampiniform plexus are dilated, with a caliber > 3 mm-although with echoic material characteristic of intraluminal thrombi^(^^6^^)^, as can be seen in Figure 9.

### Trauma

**Figure 10:**
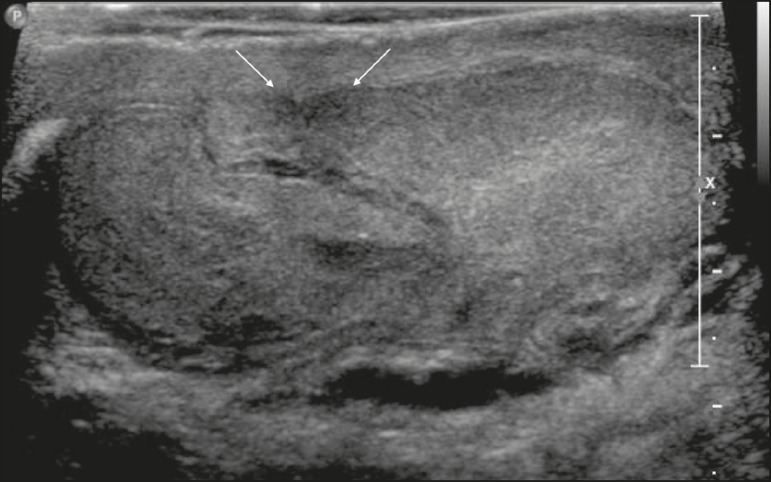
Testicular trauma. Post-trauma ultrasound of the testis showing a fracture line in the tunica albuginea (arrows). Note the diffusely heterogeneous echotexture, with hypoechoic foci of permeation, indicative of intratesticular hematoma.

Another common cause of acute testicular pain is trauma. Although the clinical history is often sufficient for making the diagnosis, ultrasound plays a central role in the evaluation of suspected testicular rupture and is indicated if there is a break in the continuity of the testicular surface, which is demarcated by the tunica albuginea. In all cases of blunt force testicular trauma, ultrasound is indicated^(^^7^^)^. If the tunica albuginea is not intact, surgery should be performed promptly in order to save the organ. The same applies if there is extruded material or loss of testicular homogeneity^(^^8^^,^^9^^)^, as depicted in Figure 10. Ultrasound is also crucial in the follow-up of cases of trauma with hematocele, because blood collections can lead to a pressure increase within the tunica, generating ischemic injury and subsequent testicular atrophy^(^^7^^,^^10^^)^, as well as increasing the risk of subsequent infection.

### Fournier's gangrene

**Figure 11:**
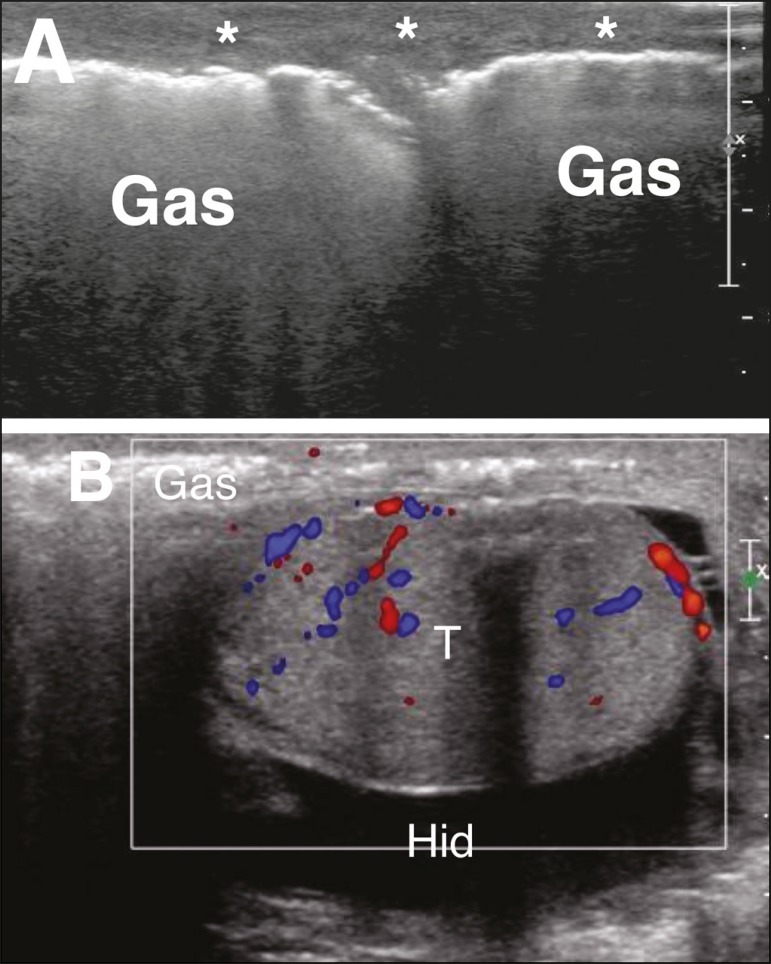
**A:** Pronounced thickening and heterogeneity of the scrotal wall and soft tissues adjacent to the testes (asterisks) extending to the perineum and containing areas of gas. The transition between the normal and thickened subcutaneous tissue is clearly seen in the image of the left testis. **B:** The testis (T) was preserved, with only a small reactive hydrocele (Hid).

Necrotizing infection of the fasciae of the perineum, commonly with gas-producing organisms, is known as Fournier's gangrene. Ultrasound can reveal involvement of the soft tissues of the scrotum, showing hyperechoic areas with acoustic shadowing indicative of gas. Although the blood supply to the walls and adjacent soft tissues of the scrotum is provided by branches of the pudendal arteries, the testes are supplied by direct branches of the aorta, explaining the fact that Fournier's gangrene often spares the testes^(^^11^^,^^12^^)^. When there is testicular involvement, the infectious focus is usually retroperitoneal or intra-abdominal^(^^11^^)^, as shown in Figure 11.

### Other infections

**Figure 12:**
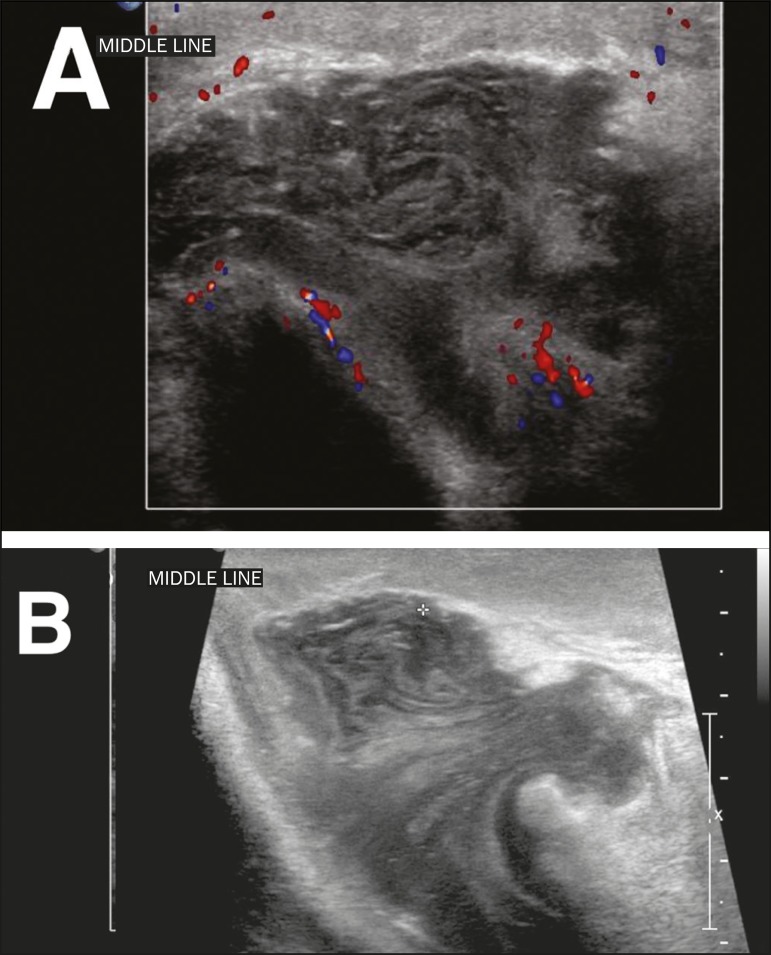
Markedly thickened and hyperechoic scrotal wall, indicating an inflammatory/infectious process, with a heterogeneous liquid collection (probable abscess) near the base of the penis.

Within the spectrum of infections of the inguinal region and scrotum is cellulitis, in which the process is limited to the subcutaneous tissue, showing hyperechogenicity and thickening, together with thin hypoechoic laminae, characteristic of exudate. Doppler ultrasound can show increased local blood flow^(^^13^^)^. In some cases, there can be abscess formation, which creates purulent collections, seen on ultrasound as hypoechoic lesions with irregular borders, with or without a surrounding halo. The interior of such an abscess is necrotic, with no vascularity seen on Doppler flow studies, whereas the periphery shows hypervascularity^(^^10^^)^, as can be seen in Figure 12.

### Testicular prostheses

**Figure 13:**
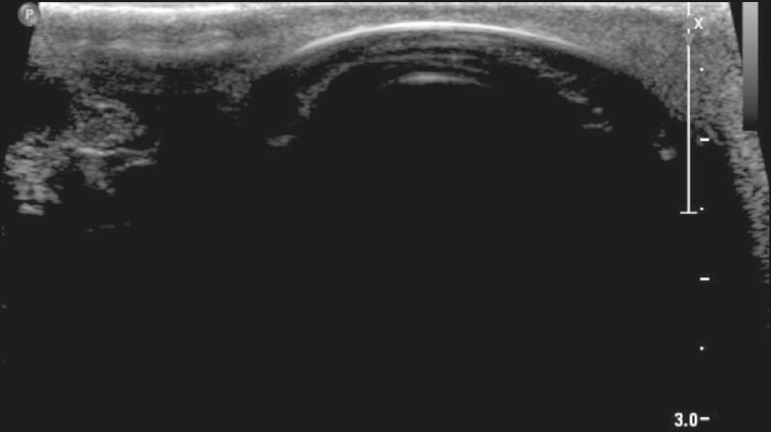
Some patients undergoing orchiectomy may opt for testicular prosthesis placement. Although the existence of a testicular prosthesis can be discovered during the anamnesis, that information is not always available. Therefore, the ultrasound technician must be able to recognize such prostheses, which present as anechoic ovoid structures.

Testicular prostheses appear as anechoic ovoid structures (Figure 13), which should be recognized by the ultrasound technician in order to avoid possible confusion.

## CONCLUSION

Ultrasound is the method of choice for the initial evaluation of the inguinal region, its main advantages being its low cost, good accuracy, and wide availability. It is important to use B mode ultrasound imaging, together with color and spectral Doppler ultrasound, and to make a comparison with the contralateral side, the correlation with the clinical history and physical examination findings being fundamental. The examiner should be familiar with the imaging aspects of the different conditions affecting this region, making an accurate, concise report, facilitating the decision-making process and consequently increasing the chance of therapeutic success.
